# Predictive Factor of Large‐Volume Central Lymph Node Metastasis in Clinical N0 Papillary Thyroid Carcinoma Patients Underwent Total Thyroidectomy

**DOI:** 10.3389/fonc.2021.574774

**Published:** 2021-05-19

**Authors:** Jianhao Huang, Muye Song, Hongyan Shi, Ziyang Huang, Shujie Wang, Ying Yin, Yijie Huang, Jialin Du, Sanming Wang, Yongchen Liu, Zeyu Wu

**Affiliations:** ^1^Department of General Surgery, Guangdong Provincial People’s Hospital, Guangdong Academy of Medical Sciences, Guangzhou, China; ^2^Shantou University Medical College, Shantou University, Shantou, China; ^3^School of Medicine, South China University of Technology, Guangzhou, China; ^4^The Second School of Clinical Medicine, Southern Medical University, Guangzhou, China

**Keywords:** central lymph node metastasis, total thyroidectomy, tumor diameter, conventional papillary thyroid cancer, capsule invasion

## Abstract

Large‐volume central lymph node metastasis (large-volume CLNM) is associated with high recurrence rate in papillary thyroid carcinoma (PTC) patients. However, sensitivity in investigating large-volume CLNM on preoperative ultrasonography (US) is not high. The aim of this study is to investigate the clinical factors associated with large-volume CLNM in clinical N0 PTC patients. We reviewed 976 PTC patients undergoing total thyroidectomy with central lymph node dissection during 2017 to 2019. The rate of large-volume LNM was 4.1% (40 of 967 patients). Multivariate analysis showed that male gender and young age (age<45 years old) were independent risk factors for large-volume CLNM with odds ratios [(OR), 95% confidence interval (CI)] of 2.034 (1.015-4.073) and 2.997 (1.306–6.876), respectively. In papillary thyroid microcarcinoma (PTMC), capsule invasion was associated with large-volume CLNM with OR (95% CI) of 2.845 (1.110–7.288). In conventional papillary thyroid cancer (CPTC), tumor diameter (>2cm) was associated with large-volume CLNM, with OR (95% CI) 3.757 (1.061–13.310), by multivariate analysis. In ROC curve analysis on the diameter of the CPTC tumor, the Area Under Curve (AUC) =0.682(p=0.013), the best cut-off point was selected as 2.0cm. In conclusion, male gender and young age were predictors for large-volume CLNM of cN0 PTC. cN0 PTMC patient with capsule invasion and cN0 CPTC patient with tumor diameter >2cm were correlated with large-volume CLNM. Total thyroidectomy with central lymph node dissection may be a favorable primary treatment option for those patients.

## Introduction

Papillary thyroid cancer (PTC) is the most common type of differentiated thyroid cancer, which account for 85%-90% of all cases (1). The incidence of PTC has been rapidly increasing globally because of early detection (2). According to the World Health Organization (WHO), PTMC is defined as PTC with maximum diameter of 10mm. Those with maximum diameter of more than 10mm is called CPTC (3).

PTC has a high metastasis rate especially central lymph node metastasis (CLNM), approximately 30%-80% of PTC patients have CLNM (4). It has been reported that 30% to 65% of patients with cN0 PTC are detected CLNM (5). The recurrence rate of patients with CLNM mainly depends on the number of involved lymph node and the size of the largest lymph node (6). Patients with large-volume lymph node metastasis (>5 involved lymph nodes) can significantly increase the risk of recurrence during long-time survival, associated with 20% recurrence rates, while small-volume metastasis (≤5 involved lymph nodes) accounts for 5% recurrence rates (7).

In the previous study, young age and male sex have been highlighted with large-volume CLNM in PTMC patient, suggesting surgery may be the primary option for young male patients (8). PTMC with multifocality, tumor diameter>0.5cm and extrathyroidal extension (ETE) also tends to have large-volume CLNM (9, 10). However, the clinicopathologic factors associated with large-volume CLNM in CPTC are not well-established (11). Sensitivity of ultrasound examination for central neck lymph node metastasis was not high (12). Therefore, identifying risk factors for large-volume CLNM in clinical N0 PTC patients might be helpful in deciding on a management strategy in PTC patients.

## Materials and Methods

### Study Population

A total of 976 PTC patients who received primary total thyroidectomy with central lymph node dissection treatment in the Department of General Surgery at Guangdong General Hospital between October 2017 to September 2019 were reviewed retrospectively in this study. The study was approved by Research Ethics Committee of Guangdong Provincial People’s Hospital, Guangdong Academy of Medical Sciences. All patients gave their informed consent to the collection of data according to the local ethic committee indications. Ultrasound examination (US) was routinely performed to assess the thyroid and lymph node status in all these patients. Computed tomography scan (CT) and positron emission tomography-computed tomography (PET-CT) were used only in some patients as needed. Patients were diagnosed PTC with LNM by pathological examination in the Pathology Department. LNM was defined as preoperatively cN1 with following signs in preoperative ultrasound examination: the ration of transverse/long diameter in cervical lymph node >0.5, blurred corticomedullary boundary, vanished medulla structure, microcalcification or cystic changes (13). Patients were excluded if they exhibited the following criteria (I) pathologic-confirmed not PTC; (II) met preoperatively cN1 diagnostic criteria; (III) received prior surgery or radiotherapy of the neck. (IV) confirmed distant metastasis or gross extrathyroidal extension.

After excluding 612 patients with suspected CLNM preoperatively, 364 patients were involved. Gender, age, ultrasound features and pathologic characteristics are recorded. Patients were divided into two groups by age: (I) age<45 years old; (II)age≥45 years old. In cases of multifocal PTCs, the diameter of the largest tumor was used. ETE only accounted for microscopic extension of tumors. The diagnosis of autoimmune thyroid disease and nodule goiter was determined by general pathological examination.

### Statistical Analysis

Statistical analysis was performed with IBM SPSS statistics 26.0 software. The relationships between large-volume LNM and clinicopathologic characteristics were investigated by the X^2^ test or one-way ANOVA to test univariate analyses. The multivariate analysis was performed on the variables that achieved P <0.05 in the univariate analysis and predictive factors for large-volume LNM were tested by the logistic regression analysis and the data was presented as the mean ± SD. P values< 0.05 (two sided) were considered statistically significant.

## Results

### Clinicopathological Characteristics of the Study Patients

The clinicopathological characteristics of the 364 study patients according to CLNM status are listed in **Table 1**. Among these, 324 (89.0%) had small‐volume CLNM, and 40 (11.0%) had large-volume CLNM. The mean age was 41.02 ± 11.324. The numbers of patients were 220 (60.4%) and 144 (39.6%) in Groups I‐II, respectively. 235 patients were female (64.6%), and 35.4% (129 patients) were male. 232 (63.7%) patients had PTMC, while 132 (36.3%) patients had tumors larger than 1cm. With the pathology examination, a total of 66 (18.1%) patients were diagnosed with Hashimoto’s thyroiditis and 93 (25.5%) patients had nodular goiter. 87 (23.9%) patients had capsule invasion and 56 (15.3%) patients had ETE.

**Table 1:** Clinicopathological characteristics and univariate analysis of PTC patients (n=364).

	Small-volume (n=324)	Large-volume (n=40)	p-value
Gender			0.017*
Male	108	21	
Female	216	19	
Age			0.046*
<45	190	30	
≥45	134	10	
Bilateral tumors			0.094
Bilateral	76	14	
Unilateral	248	26	
Multifocal tumor			0.754
Multifocal	103	18	
single	221	22	
Hashimoto’s thyroiditis			0.157
Yes	62	4	
No	262	36	
Nodule			
Goiter			0.049*
Yes	87	5	
No	237	35	
Marked Hypoechoic			0.745
Yes	266	32	
No	58	8	
Margin			0.207
Irregular	276	31	
Regular	48	9	
Shape			0.167
Well-defined	46	9	
Non-well-defined	278	31	
Capsule Invasion			0.003*
Yes	70	17	
No	254	23	
ETE**			0.001*
Yes	43	13	
No	281	27	

*statistically significance; **extrathyroidal extension.

### Risk Factors for Large-Volume CLNM in PTC

In univariate analysis, young age (<45 years), male, multifocality, nodule goiter and ETE were significantly associated with a high prevalence of large-volume CLNM (**Table 1**). However, bilateral tumors, marked hypoechoicity, irregular margin and non-well-defined shape were not correlated with the large-volume CLNM. In multivariate analysis, young age [odds ratio (OR): 2.034, 95% confidential interval (CI): 1.015-4.073, P =0.045] and male (OR: 2.997, 95% CI: 1.306–6.876, P= 0.010) were still significant predictive factors for large-volume CLNM (**Table 2**).

**Table 2:** Multivariate analysis of the risk factors of PTC.

	Sig.	OR (95% CI)
Gender	0.045	2.034 (1.015-4.073)
Age	0.010	2.997 (1.306-6.876)
Capsule Invasion	0.284	0.561 (0.194-1.616)
ETE*	0.136	0.408 (0.125-1.326)
Nodule goiter	0.111	2.254 (0.829-6.130)

*extrathyroidal extension.

### Risk Factors for Large-Volume CLNM in PTMC and CPTC

**Figure 1:**
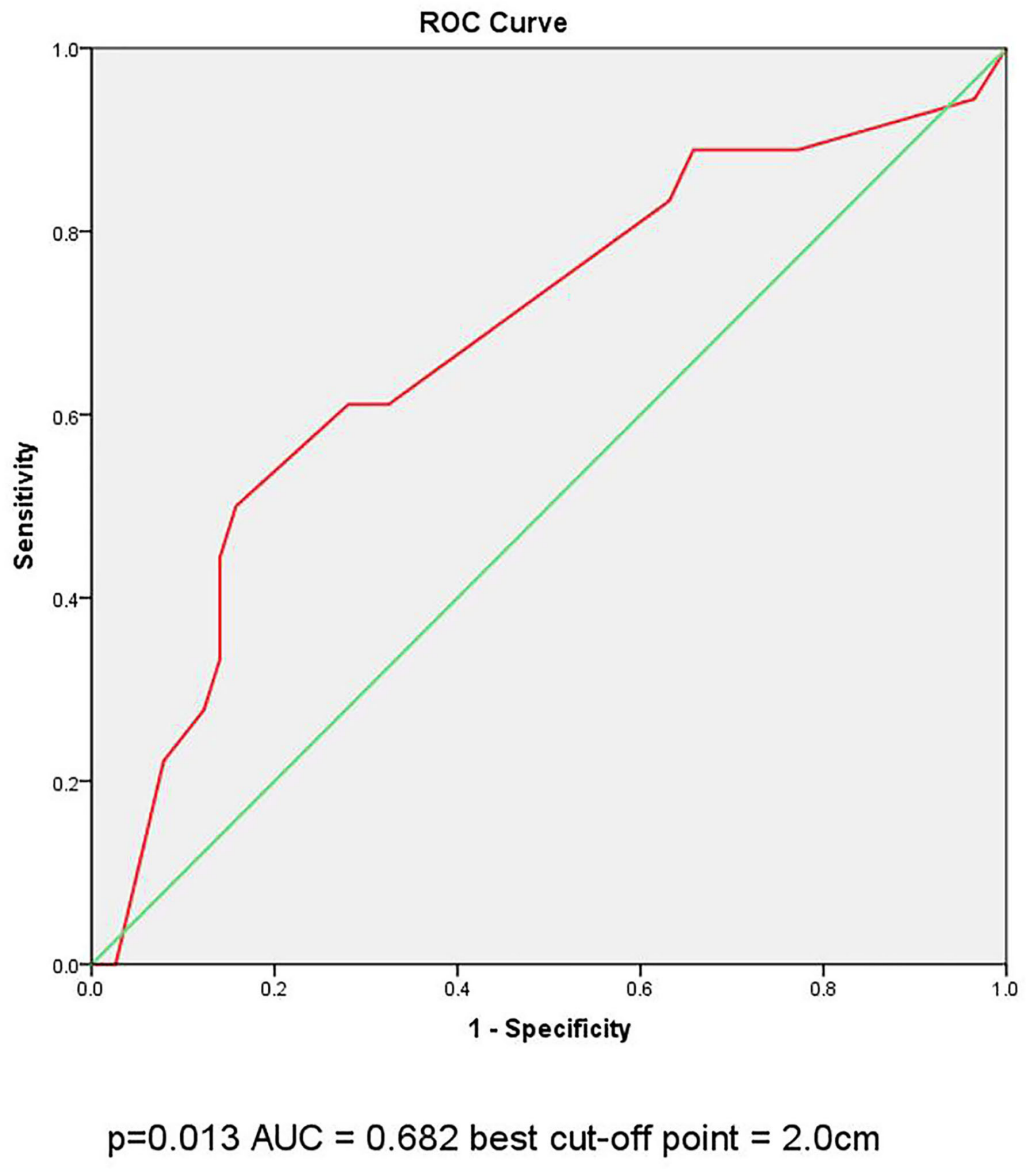
ROC curve analysis of the cutoff point for diameter of the CPTC tumor.

Divided by the maximum diameter of the tumor, clinicopathologic characteristic were tested in PTMC patients and CPTC patients, respectively. In 232 PTMC patients, male, multifocality, nodule goiter and capsule invasion were significantly correlated with large-volume CLNM (**Table 3**). Age and ETE were not correlated with the large-volume CLNM. In multivariate analysis, shown in **Table 4**, only capsule invasion (OR: 2.845, 95% CI: 1.110–7.288, P= 0.029) was still significant predictive factors for large-volume CLNM. In 132 CPTC patients, Hashimoto’s thyroiditis, marked hypoechoicity, irregular margin and tumor diameter (>2cm) were significantly correlated with large-volume CLNM (**Table 5**). Age and gender were not correlated with the large-volume CLNM. In multivariate analysis, shown in **Table 6**, tumor diameter (>2cm) (OR: 3.757, 95% CI: 1.061–13.310, P= 0.04) was significant predictive factors for large-volume CLNM. The high sensitivities and low false-negative rates (1- specificity) associated with the tumor diameter were identified *via* ROC curve analysis, as depicted in **Figure 1**. The Area Under Curve (AUC) =0.682(p=0.013), the best cut-off point was selected as 2.0 cm, which meant that CPTC patients with tumor diameter higher than 2.0cm had more likelihood to have large-volume CLNM.

**Table 3:** Clinicopathological characteristics and univariate analysis of PTMC patients (n=232).

	Small-volume (n=210)	Large-volume (n=22)	p-value
Gender			0.028*
Male	74	13	
Female	136	9	
Age			0.184
<45	122	16	
≥45	88	6	
Bilateral tumors			0.292
Bilateral	46	7	
Unilateral	164	15	
Multifocal tumor			0.043*
Multifocal	61	11	
single	149	11	
Hashimoto’s thyroiditis			0.964
Yes	39	4	
No	171	18	
Nodule			0.226
Goiter			
Yes	53	3	
No	157	19	
Margin			0.748
Irregular	179	20	
Regular	31	2	
Shape			1.00
Well-defined	30	3	
Non-well-defined	180	19	
Marked Hypoechoic			0.115
Yes	173	21	
No	37	1	
Invasion			0.004*
Yes	40	12	
No	170	10	
ETE**			0.079
Yes	28	6	
No	182	16	
Size of tumors			0.916
>0.5	136	14	
≤0.5	74	8	

*statistically significance; **extrathyroidal extension.

**Table 4:** Multivariate analysis of the risk factors of PTMC.

	Sig.	OR (95% CI)
Gender	0.123	2.080 (0.819-5.281)
Multifocality	0.121	2.064 (0.826-5.160)
Capsule Invasion	0.029	2.845 (1.110-7.288)

**Table 5:** Clinicopathological characteristics and univariate analysis of CPTC patients (n=132).

	Small-volume (n=114)	Large-volume (n=18)	p-value
Gender			0.216
Male	34	8	
Female	80	10	
Age			0.141
<45	68	14	
≥45	46	4	
Bilateral tumors			0.270
Bilateral	30	7	
Unilateral	84	11	
Multifocal tumor			0.754
Multifocal	40	7	
single	74	11	
Hashimoto’s thyroiditis			0.042*
Yes	19	0	
No	91	18	
Nodule			
Goiter			0.406
Yes	34	16	
No	80	2	
Marked Hypoechoic			0.048*
Yes	93	11	
No	12	7	
Margin			0.022*
Irregular	97	11	
Regular	17	7	
Shape			0.08
Well-defined	16	6	
Non-well-defined	98	12	
Capsule Invasion			0.27
Yes	30	7	
No	84	11	
ETE**			0.013*
Yes	15	7	
No	99	11	
Size of tumors			0.005*
>2cm	16	8	
≤2cm	98	10	

*statistically significance; **extrathyroidal extension.

**Table 6:** Multivariate analysis of the risk factors of CPTC.

	Sig.	OR (95% CI)
Hashimoto’s Thyroiditis	0.998	0.00
Margin	0.596	0.649 (0.131-3.219)
Shape	0.419	0.533 (0.116-2.452)
Diameter	0.040	3.757 (1.061-13.310)
ETE*	0.072	3.194 (0.900-11.332)

*extrathyroidal extension.

## Discussion

Previous studies have demonstrated that male sex, age, multifocality, tumor diameter>0.5cm and ETE were independent risk factors for LNM in PTMC patients (8–10). In this study, univariate and multivariate analyses revealed that age <45 years and male gender are independent predictive factors for large-volume CLNM in cN0 PTC patients. In addition, capsule invasion is independent predictive factors for large-volume CLNM in cN0 PTMC, while tumor diameter >2cm predicts large-volume CLNM in in cN0 CPTC.

Age is regarded as the most important prognostic factors for thyroid cancer. 45 years is widely adopted as clinically important prognostic marker in PTC patients. Our study demonstrates that the rate of large-volume CLNM was higher in patients < 45 years than that ≥45 years (13.6% *vs.* 6.9%, P = 0.046). Multivariate analysis shows age < 45 years was independent predictor of large-volume CLNM inpatients with cN0 PTC. Similarly, previous studies have reported that CLNM was more likely to appear in PTC patients with age < 45 years, which may be due to rapid tumor diameter growth in young patient during surveillance period (6, 14, 15). These results indicate that much careful preoperative assessment of the lymph node status must be followed in young patients.

Male gender has been identified as a risk factor for thyroid carcinoma (16). Similarly, recent studies have revealed that men exhibited aggressive behavior and poorer prognosis than women among PTC patients (17). Among PTC patients who did not undergo prophylactic central neck dissection, male gender is a risk factor for further recurrence (18). This study also showed male gender as the predictive factor for large-volume CLNM in cN0 PTC. Therefore, male patient with cN0 PTC should be evaluated carefully and complete central neck dissection may be favorable.

With the increased diagnosis of PTC, a growing number of patients with PTMC have been detected. PTMC appears to account for the majority of the increased incidence of thyroid cancer (19). Capsule invasion is considered as aggressive behavior of PTMC, which is correlated with CLNM, even with lymph nodes posterior to right recurrent laryngeal nerve metastasis (LN-prRLN) (14, 20). In PTMC, previous studies did not demonstrate the clear relationship between capsule invasion and large- volume metastasis. A novel finding in our study is that capsule invasion is more likely to present with large-volume metastasis. (P=0.029, OR =2.845, with 95% CI: ranged from 1.110-7.288). The incidence of large-volume CLNM in capsule invasion and non-capsule invasion were 23% *vs.* 5.6%, with a P value <0.05 in multivariate analysis. It might be expected that the PTMC with capsule invasion more likely showed extrathyroidal metastasis potential to lymph node and distant organs, which significantly accounted for the high incidence of large-volume CLNM and high recurrence rate.

Tumor size is considered as an important factor in TNM staging for PTC, and larger tumors tend to be more aggressive (21, 22). Recent studies reported that tumor diameter >0.5 cm was independent risk factors for large-volume CLNM in cN0 PTMC (8, 9). However, Shen et al. (10) demonstrated that the tumor diameter >0.5 cm was not significantly different between the large-volume CLNM group and non- large-volume CLNM volume group. We were also unable to investigate the relationship between tumor diameter and large volume metastasis in cN0 PTMC. However, in CPTC, we found that tumor diameter higher than 2cm is associated with large-volume CLNM. Ito et al. (23) and Ma et al. (14) reported that tumor diameter of >2cm is the strongest predictor of CLNM and lymph node recurrence in PTC. Compared with tumor diameter <2cm, tumor diameter >2cm was correlated with the five times higher risk of recurrence in PTC patients aged ≥55 years old (24). ROC curve analysis was used to determine the cutoff point of tumor size for predicting large-volume metastasis and found that tumor diameter higher than 2cm was the strongest predictor of large-volume CLNM in cN0 CPTC. Therefore, tumor diameter higher than 2cm should be evaluated carefully for possible large-volume CLNM of PTC. In addition, careful prophylactic central node dissection should be recommended for N0 CPTC with a large tumor diameter (tumor diameter >2cm).

In our study, ultrasound features are analyzed to study the correlation with large-volume CLNM in PTMC and CPTC. However, no predictive factor is established. The possible reason is that ultrasonography in investigating CLNM is not sensitive and ultrasonography diagnosis is subjective, mainly relying on doctor’s clinical experience (25, 26). In presence studies, radiomics and deep machinery learning are gradually conducted to investigate the CLNM. The highest sensitivity has reach 0.858 in the existing predictive models of CLNM, which is significantly higher than the current situation (lower than 0.5 for predicting CLNM) (27). It may provide more dependable evidence for performing central lymph node dissection.

However, the present study has several limitations. The risk factors we identified were based on our retrospective design and the relatively small number of patients with large-volume CLNM, and thus may need further exploration. In addition, our study has no information about follow-up evaluation of the patients for potential development of recurrence and future distant metastases.

## Conclusion

In conclusion, age<45 years and male gender are independent predictors of large-volume CLNM metastasis in patients with cN0 PTC. Total thyroidectomy with central neck dissection may be a favorable option for surgeons when treating young male patients with cN0 PTC. Besides, cN0 PTMC patient with capsule invasion and cN0 CPTC patient with tumor diameter >2cm have higher prevalence of large-volume CLNM. Therefore, these factors should be considered by the surgeons when evaluating the risk of large-volume CLNM of PTC. Follow-up visits for post-operative patients with these pathologic characteristics should be regarded.

## Data Availability Statement

The raw data supporting the conclusions of this article will be made available by the authors, without undue reservation.

## Ethics Statement

The study was approved by Research Ethics Committee of Guangdong Provincial People’s Hospital, Guangdong Academy of Medical Sciences. All patients gave their informed consent to the collection of data according to the local ethics committee’s indications.

## Author Contributions

JH, MS, and HS designed this study. JH, MS, HS, ZH, and SJW collected the data. JH, MS, and HS analyzed the data. All authors contributed to the article and approved the submitted version.

## Funding

This work was supported by the Natural Science Foundation of Guangdong Province (No. 2020A1515010127), Scientific Research Staring Foundation for the Returned Overseas from Guangdong Provincial People’s Hospital (No. 2017x02) and Guangdong Provincial People‘s Hospital Scientific Foundation for Distinguished Young Scholars of Guangdong Province (No. KJ012019441).

## Conflict of Interest

The authors declare that the research was conducted in the absence of any commercial or financial relationships that could be construed as a potential conflict of interest.
