# Corrigendum: Predictive factor of large‐volume central lymph node metastasis in clinical N0 papillary thyroid carcinoma patients underwent total thyroidectomy

**DOI:** 10.3389/fonc.2024.1517682

**Published:** 2024-11-18

**Authors:** Jianhao Huang, Muye Song, Hongyan Shi, Ziyang Huang, Shujie Wang, Ying Yin, Yijie Huang, Jialin Du, Sanming Wang, Yongchen Liu, Zeyu Wu

**Affiliations:** ^1^ Department of General Surgery, Guangdong Provincial People’s Hospital, Guangdong Academy of Medical Sciences, Guangzhou, China; ^2^ Shantou University Medical College, Shantou University, Shantou, China; ^3^ School of Medicine, South China University of Technology, Guangzhou, China; ^4^ The Second School of Clinical Medicine, Southern Medical University, Guangzhou, China

**Keywords:** central lymph node metastasis, total thyroidectomy, tumor diameter, conventional papillary thyroid cancer, capsule invasion

In the published article, there was an error in the **Funding** statement. The **Funding** statement for the Guangdong Basic and Applied Basic Research Foundation was displayed as “Natural Science Foundation of Guangdong Province (No. 2020A1515010127)”. The correct statement is “Guangdong Basic and Applied Basic Research Foundation (No. 2020A1515010126)”. The correct **Funding** statement appears below.

“This work was supported by Guangdong Basic and Applied Basic Research Foundation (No. 2020A1515010126), Scientific Research Staring Foundation for the Returned Overseas from Guangdong Provincial People’s Hospital (No. 2017x02) and Guangdong Provincial People’s Hospital Scientific Foundation for Distinguished Young Scholars of Guangdong Province (No. KJ012019441).”

The authors apologize for this error and state that this does not change the scientific conclusions of the article in any way. The original article has been updated.

